# EFFICACY OF THE RESIDENTIAL CARE TRANSITION MODULE

**DOI:** 10.1093/geroni/igae098.1255

**Published:** 2024-12-31

**Authors:** Joseph Gaugler, Robyn Birkeland, Elizabeth Albers, Colleen Peterson, Katie Louwagie, Zachary Baker, Mary Mittelman, David Roth

**Affiliations:** University of Minnesota, Minneapolis, Minnesota, United States; University of Minnesota, Minneapolis, Minnesota, United States; University of Minnesota, Minneapolis, Minnesota, United States; University of Michigan Transportation Research Institute, Ann Arbor, Michigan, United States; University of Minnesota, Minneapolis, Minnesota, United States; Arizona State University, Phoenix, Arizona, United States; New York University, New York, New York, United States; Johns Hopkins University, Baltimore, Maryland, United States

## Abstract

“This study aimed to evaluate the efficacy of The Residential Care Transition Module (RCTM), a six-session, psychosocial, and psychoeducational telehealth intervention for family caregivers of cognitively impaired relatives living in a residential long-term care (RLTC) setting. Eligible participants (including care recipients, regardless of time since admission) were randomized to treatment or usual care control conditions. Survey data were collected at baseline, four months, eight months, and 12 months (N = 240). Primary analytic outcomes included caregiver subjective stress (a stress process mechanism) and depressive symptoms (a measure of global well-being). Secondary analytic outcomes included secondary role strains, residential care stress, caregiver sense of competence, and self-efficacy (additional mechanisms of action). General linear models tested for the main effects of the intervention at four months and longitudinal mixed models examined the 12-month effects of the intervention. Post hoc analyses also examined the influence of moderators. There were no significant differences between the treatment and control groups for any primary analytic outcome. Caregivers in the treatment group whose relatives were admitted to RLTC in the prior three months were more likely to indicate reductions in depressive symptoms over the first four months of participation. Over the 12-month study period, caregivers in the treatment group who were employed reported increased self-efficacy over time. The heterogeneity of dementia care requires a broader consideration of key contextual factors. Aligning measures with the preferences, goals, and values of dementia caregivers may further demonstrate the benefits of interventions such as the RCTM” (Gaugler et al., in press).

